# The Treatment of Venereal Diseases, a Monograph on the Method Pursued in the Vienna Hospital

**Published:** 1872-07

**Authors:** 


*The Treatment of Venereal Diseases.* A Monograph on the Method pursued in the Vienna Hospital, under the direction of Prof. Von Sigmund; including all the Formulæ. By M. H. HENRY, M. D. New York: Wm. Wood & Co. 1872.

The Venereal Department of the Vienna Hospital is noted for its admirable arrangement and the excellent oportunities for clinical study which it offers.

We know of no one more adapted to present to English readers the Vienna system of practice, than Dr. Henry the Editor of the American Journal of Syphilography and Dermatology; whose cultivation of this branch of medicine has made him an authority on venereal diseases.

In speaking of the department of venereal diseases in the Vienna Hospital, and the course there pursued, the authors says:

"It results that a *resume* of experience in this establishment cannot fail greatly to extend our own knowledge and afford more reliable data and inductions in the treatment of this class of diseases in this city,—for *experientia docet* was assuredly never more applicable than in venereal therâpeutics.

To the practitioner the minute details of treatment will be of especial interest and value; while the numerous formulæ (including about two hundred) afford a liberal choice in the management of these affections."

